# Beneficial Effects of the mTOR Inhibitor Everolimus in Patients with Advanced Medullary Thyroid Carcinoma: Subgroup Results of a Phase II Trial

**DOI:** 10.1155/2015/348124

**Published:** 2015-07-29

**Authors:** T. C. Schneider, D. de Wit, T. P. Links, N. P. van Erp, J. J. M. van der Hoeven, H. Gelderblom, T. van Wezel, R. van Eijk, H. Morreau, H. J. Guchelaar, E. Kapiteijn

**Affiliations:** ^1^Department of Medical Oncology, Leiden University Medical Center, 2333 ZA Leiden, Netherlands; ^2^Department of Pathology, Leiden University Medical Center, 2333 ZA Leiden, Netherlands; ^3^Department of Clinical Pharmacy and Toxicology, Leiden University Medical Center, 2333 ZA Leiden, Netherlands; ^4^Department of Endocrinology, University Medical Center Groningen, 9700 RB Groningen, Netherlands; ^5^Department of Clinical Pharmacy, Radboud University Nijmegen Medical Center, 6525 GA Nijmegen, Netherlands

## Abstract

*Objective*. Until recently, advanced medullary thyroid cancer (MTC) had few treatment options except surgery. The mTOR inhibitor everolimus has shown encouraging results in neuroendocrine tumors. As part of a prospective phase II study, we analyzed the safety and efficacy of everolimus in advanced MTC. *Methods*. Seven patients with per RECIST 1.1 documented advanced MTC were included and received everolimus 10 mg daily. The primary objective was determining treatment efficacy. Secondary endpoints included progression-free survival (PFS), overall survival (OS), toxicity, and pharmacokinetics (PK). *Results*. Median follow-up duration was 28 weeks (17–147). Five patients (71%) showed SD, of which 4 (57%) showed SD >24 weeks. Median PFS and OS were 33 (95%CI: 8–56) and 30 (95%CI: 15–45) weeks, respectively. Toxicity was predominantly grade 1/2 and included mucositis (43%), fatigue (43%), and hypertriglyceridemia (43%). Four MTCs harbored the somatic *RET* mutation c.2753T>C, p.Met918Thr. The best clinical response was seen in a MEN2A patient. PK characteristics were consistent with phase I data. One patient exhibited extensive toxicity accompanying elevated everolimus plasma concentrations. *Conclusions*. This study suggests that everolimus exerts clinically relevant antitumor activity in patients with advanced MTC. Given the high level of clinical benefit and the relatively low toxicity profile, further investigation of everolimus in these patients is warranted.

## 1. Introduction

Medullary thyroid cancer (MTC) is a neuroendocrine tumor derived from the calcitonin-producing thyroid C cells and accounts for 3–5% of cases of thyroid cancer [1]. Until recently, besides surgery few curative and palliative treatment options were available for patients with MTC. Conventional treatment modalities have disappointing outcomes in most patients with MTC, indicating the need for new treatments.

The American Thyroid Association therefore recommends that these patients should be enrolled in clinical trials. Concurrently, an increased understanding of thyroid tumorigenesis has led to the identification of potential targets, with novel therapeutic agents now able to target these biological abnormalities.

In addition, many genetic alterations affecting tyrosine kinase signaling pathways have recently been identified in various forms of thyroid cancer such as the activating* RET* mutations present in >95% of hereditary MTC and in 20–50% of sporadic MTC [2, 3]. The phosphatidylinositol-3-kinase (PI3K)/Akt pathway regulates cell growth, proliferation, and survival in all thyroid tumor subtypes [4]. An important effector in the PI3K/Akt pathway is the mammalian target of rapamycin (mTOR). Activation of the mTOR serine/threonine protein kinase has been reported in a variety of malignant tumors, including thyroid tumors, with an estimated 70% of all tumors showing mTOR upregulation [5].

As a result of this accrued understanding of the biological basis of thyroid cancer development, several clinical trials with multitarget tyrosine kinase inhibitors (TKIs) have been conducted. Everolimus is an orally available derivative of rapamycin, targeting mTOR. Everolimus exerts its activity through high affinity interaction with an intracellular receptor protein, the immunophilin FKBP12. The FKBP12/everolimus complex subsequently interacts with the mTOR protein kinase, inhibiting downstream signaling events involved in regulation of the G1 to S-phase transition [6]. The use of everolimus in neuroendocrine tumors has shown encouraging results, both in vitro and in vivo [7–9].

We initiated a phase II study to assess safety and efficacy of everolimus on tumor progression in patients with advanced thyroid carcinoma (THYRRAD, www.clinicaltrials.gov  CRAD001CNL08T). In addition, pharmacokinetic parameters were assessed. Here we present a subgroup analysis of seven MTC patients.

## 2. Materials and Methods

### 2.1. Patients

Eligibility criteria were the presence of per RECIST 1.1 (Response Evaluation Criteria in Solid Tumors) documented progressive metastatic or inoperable MTC in the 12 months prior to therapy [10]. Patients were required to be ≥ 18 years of age with a Karnofsky performance score > 70%. Laboratory requirements consisted of adequate bone marrow function (absolute neutrophil count ≥ 1,500/*μ*L, platelets ≥100,000/*μ*L, and hemoglobin ≥ 5.6 mm/L), liver function (serum bilirubin ≤ 1.5x upper limit of normal (ULN) and serum ALT and AST ≤ 2.5x ULN), and renal function (serum creatinine ≤ 2x ULN). Women of childbearing age were required to have a negative serum or urinary pregnancy test within 14 days prior to the first dose of the study drug. Patients were not excluded based on number or type of prior therapies received, with the exception of prior targeted therapy with everolimus or other mTOR inhibitors.

Written informed consent was provided by all patients before enrollment in the trial. The study protocol was approved by the Institutional Review Board of Leiden University Medical Center and performed in the university hospitals of Leiden and Groningen. This study was registered at ClinicalTrials.gov (#NCT01118065).

### 2.2. Study Design

We performed a nonrandomized, open-label, multicenter, single arm phase II trial of everolimus in patients with advanced thyroid cancer; 28 patients with differentiated (DTC), 7 patients with anaplastic (ATC), and 7 patients with medullary (MTC) thyroid carcinoma were included. The primary objective was to determine the efficacy (response rate and stable disease >24 weeks) of everolimus. Secondary objectives were determination of the maximum percentage of tumor reduction for target lesions, describing activity time to event endpoints, and assessment of toxicity, adverse events (AEs), and pharmacokinetics. Eligibility assessments, including a review of medical history and prior treatments, physical examination, and disease staging assessments were performed within 4 weeks prior to the first dose of everolimus. Baseline evaluations, comprising performance status, vital signs, and laboratory tests, were assessed within 2 weeks prior to initiation of therapy. Everolimus was administered at a dose of 10 mg orally once daily until disease progression, unacceptable toxicity, death, or patients' own request. Objective tumor response and time of progressions were measured according to the RECIST criteria version 1.1 every 12 weeks (±2 weeks) during the first year, thereafter every 6 months and at study discontinuation.

Safety assessments were made every 4 weeks by adverse event collection, standard clinical and laboratory tests, and physical examinations. In case of toxicity or AEs requiring dose adjustments, drug dosing was interrupted or modified according to the guidelines. AE monitoring was continued for at least 4 weeks following the last dose of study treatment. The incidence, grade, and casual relationship of adverse events were graded with the use of Common Terminology Criteria for Adverse Events (CTCAE, version 4.0). Following study discontinuation, all patients were followed for survival for 4 weeks.

### 2.3. Laboratory Parameters

Serum thyroid stimulating hormone (TSH), free thyroxine (T4), carcinoembryonic antigen level (CEA), calcitonin, and safety parameters were assessed at all visits. Safety parameters included a total blood count as well as serum levels of sodium, potassium and creatinine, lipids, coagulation, and renal and liver function.

### 2.4. Somatic Mutation Spectrum Screening

DNA for mutation analysis was available for six MTC tumors. Somatic hotspot mutations were identified using a custom AmpliSeq panel that targets somatic hotspot mutations in 22 genes using Ion Torrent AmpliSeq sequencing chemistry (Life Technologies, Foster City, CA, details available on request). The samples were sequenced using the Ion Torrent PGM (Life Technologies, Foster City, CA). FastQ sequence data files from the Ion Torrent PGM were analyzed with NextGENe software (version v.2.3.4.2, Softgenetics, State College, PA) using standard settings for somatic mutation analysis, excluding known polymorphisms.

### 2.5. Pharmacokinetics

In order to measure blood concentration levels of everolimus and assess everolimus steady-state pharmacokinetics (PK), patients were admitted to hospital for PK sampling on day 15 of each treatment cycle. Samples were collected in EDTA tubes at predose and at 1, 2, and 3 hours after everolimus intake. Additional PK sampling at 4, 5, 6, 7, and 8 hours after everolimus intake was optional. Everolimus concentrations in whole blood were determined using a validated Ultra Performance Liquid Chromatography-Tandem Mass Spectrometric (UPLC-MS/MS) assay. Pharmacokinetic parameters were calculated with a noncompartmental approach using WinNonLin and included the area under the concentration time curve over the dosing interval (AUC_0–24 hr_), trough everolimus concentration (*C*_trough_), time to reach peak concentration (*T*_max_), peak concentration (*C*_max_), and the elimination half-life (*T*_1/2_).

### 2.6. Statistical Analysis

Seven MTC patients were analyzed as a separate cohort for response rate. If no responses were present in this patient group, we could conclude that further investigation of everolimus in MTC patients is unwarranted. Endpoints were reported as median (range) or proportions. Estimates of progression-free survival (PFS) (time from starting study drug to progression or death, whichever occurred first) and OS (time from starting study drug to the date of death by any cause), with associated 95% CIs, were obtained using the Kaplan-Meier method. Patients who were progression-free and/or alive at the time of data analysis were censored. Variables influencing the response to everolimus were analyzed with binominal logistic regression. The calculations were performed using SPSS 20.0 for Windows (SPSS, Chicago, IL, USA).

## 3. Results

### 3.1. Patient Characteristics

All 7 patients with medullary thyroid carcinoma were included in this efficacy and tolerability analysis. The follow-up of the study ended on December 31, 2013, with a median follow-up of 28 weeks (range 17–145 weeks). The median everolimus treatment period was 17 weeks (range 6–116 weeks). One patient was still on everolimus treatment at the time follow-up ended.

Baseline characteristics are listed in Table 1. Two (29%) females and 5 (71%) males were included, with a median age of 53 years (range 44–74). At study entry, 1 (14%) patient had locally advanced disease, while the other 6 (86%) had distant metastasis. None of the patients were treatment naïve.

### 3.2. Efficacy

**Figure 1:**
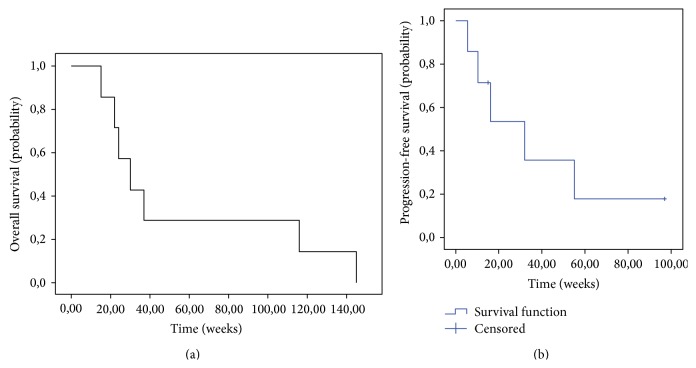
Kaplan-Meier curves of (a) overall survival and (b) median progression-free survival.

Efficacy analysis showed promising results. Five patients (71.4%) showed stable disease (SD), with 4 (57.1%) having SD lasting >24 weeks. Median SD duration was 24 weeks (range 17–117 weeks). At the time of data analysis (31-12-2013), 1 patient still had ongoing SD. There were no complete (CR) or partial (PR) responses. Data on efficacy are given in Table 2 and Figure 1.

**Figure 2:**
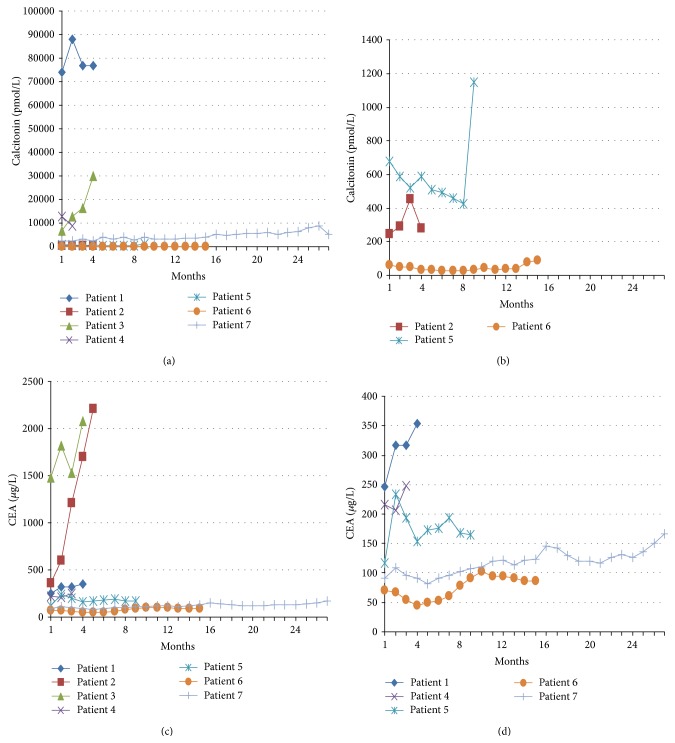
Serum calcitonin and CEA concentrations per patient over time. (a) Serum calcitonin over time for all patients, (b) close-up of figure (a) for patients 2, 5, and 6, (c) serum CEA for all patients, and (d) close-up of figure (c) for patients 1, 4, 5, 6, and 7.

Estimated median PFS was 33 weeks (95% CI: 8–56 weeks). The median overall survival (OS) was 30 weeks (95% CI: 15–45 weeks). Disease progression and survival appeared not to be influenced by age, gender, disease site, mutational status, everolimus blood concentration, or dose reduction. Changes in calcitonin and CEA could not be related to clinical outcome, as demonstrated in Table 3 and Figure 2.

### 3.3. Toxicity

Three patients (43%) required dose reduction due to toxicity. One patient (14%) discontinued the study after 12 weeks due to complaints of fatigue and peripheral edema. All observed AEs are listed in Table 4. Treatment-related AEs were predominantly grade 1 or 2, with the most common events including mucositis, fatigue, and hypertriglyceridemia. Grade 3 AEs consisted of fatigue (29%), peripheral edema (14%), hypercholesterolemia (14%), hyperglycemia (14%), pneumonia (14%), and pneumonitis (14%). No grade 4 AEs were observed. One (14%) serious adverse event (SAE) was reported when a patient was hospitalized due to acute stomach pain after 5 weeks of everolimus treatment. A gastroduodenoscopy revealed no abnormalities and the complaints resolved spontaneously. The majority of AEs were controllable with dose reduction, medication, or supporting measures. There appeared to be no relation between toxicity and performance state or age.

**Figure 3:**
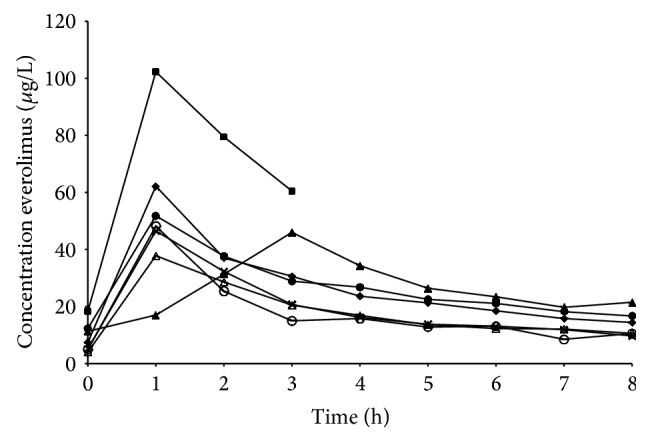
Individual observed concentration versus time profiles for everolimus.

One patient with relatively high everolimus blood plasma concentrations (AUC_0–24 hr_, 960 *μ*g*∗*hr/L), as shown in Figure 3, also showed more profound toxicity. This patient suffered a total of 13 AEs, whereas the median number of AEs was 5 (range 3–13). Comedication and a high hematocrit were excluded as possible causes of the higher plasma concentrations in this patient.

### 3.4. Mutation Analysis

One of the 7 MTC patients was excluded from the mutation analysis because no tumor tissue was available. Of the 6 remaining patients, one MEN2A patient carried a germline* RET* c.1858T>C, p.Cys620Arg mutation. Using targeted nextgen sequencing of tumors, four showed a somatic* RET* c.2753T>C, p.Met918Thr mutation, including one that also carried an* EGFR* c.2543C>T, p.Pro848Leu mutation (see supplementary Table 1 in Supplementary Material available online at http://dx.doi.org/10.1155/2015/348124). It is noteworthy that the MEN2A patient showed the best response to everolimus treatment, with the longest period of stable disease.

### 3.5. Pharmacokinetics

One patient completed only the short PK sampling schedule; all other patients participated in the extended sampling schedule up to 8 hours after everolimus intake. Individual everolimus concentrations versus time profiles are shown in Figure 3.

A summary of everolimus pharmacokinetics is shown in Table 5. The median (range) AUC_0–24 hr_ for everolimus was 421 *μ*g*∗*hr/L (257–960 *μ*g*∗*hr/L) and the median *C*_trough_ was 7.4 *μ*g/L (4.0–18.3 *μ*g/L). The median *T*_max_ was 1.0 hour and the median *C*_max_ was 48.2 *μ*g/L. The median *T*_1/2_ of everolimus was 13.8 hours (10.9–32.4 hr).

## 4. Discussion

Until recently, surgery was accompanied by only limited curative and palliative treatment options for patients with MTC, emphasizing the need for new therapies. Using everolimus, a significant dose-dependent inhibition in cell proliferation was observed in two medullary thyroid cancer cell lines [11]. Everolimus significantly inhibited cell viability in a dose- and time-dependent fashion and diminished phosphorylation of mTOR in a TT thyroid cancer cell line and cultured human MTCs [12]. Two case reports comprising three patients with advanced MTC showed beneficial effects of everolimus [11, 13]. Recently, a phase II study in patients treated with everolimus, with advanced thyroid cancer of all histologic subtypes (*n* = 38), reported a partial response (PR) and stable disease (SD) in 5% and 76% of patients, respectively. Median progression-free survival (PFS) was 47 weeks [14].

Our phase II trial on advanced MTC showed promising results, with stable disease in 5 of 7 (71%) patients. The median PFS and OS of 33 and 30 weeks, respectively, were also promising. The lack of PRs is easily explained by the fact that these tumors are slowly progressive and PRs were therefore not expected. Due to the fact that only seven patients were included, we were not able to identify parameters significantly influencing response or survival. Although several trials have reported a biochemical CEA and calcitonin response that reflects the radiological response, these changes were not always significant in our study and we were unable to confirm this correlation [11, 14–16].

Although EGFR overexpression is frequently seen in medullary thyroid cancer, EGFR mutations are rarely described [17]. The* EGFR* P848L mutation we identified is situated close to the L858R mutation, a well-known target in nonsmall cell lung cancer. However, the P848L mutation has been described in patients with nonsmall cell lung carcinoma and is thought to be a functionally silent polymorphism, insensitive to gefitinib treatment [18, 19].

The MEN2A MTC patient showed the best response upon everolimus therapy. Apart from the treatment effect, the* RET* c.1858T>C, p.Cys620Arg mutation has been associated with a less aggressive phenotype compared to other MEN2A-related mutations such as the c.2753T>C p.Met918Thr mutation or one of the classic mutations in exon 11 at codon 634 [20]. To the best of our knowledge, this is the first report of administration of everolimus to a MEN2A patient.

Everolimus was generally well tolerated, with the majority of AEs being manageable and similar to previously reported toxicities [14, 21]. The PK characteristics were also consistent with phase I pharmacokinetic studies investigating everolimus (10 mg once daily) in solid and hematological malignancies [22–24]. The interpatient variability in PK was also comparable to previous research. Although based on the short sampling schedule, the patient with the highest exposure also showed the most extensive toxicity profile, perhaps related to higher everolimus plasma concentrations.

Although everolimus treatment has shown encouraging results in patients with a variety of solid tumors, it has to be noted that its inability to inhibit mTORC2 may imply an inability to achieve a potent and long term antitumor effect. Hisamatsu et al. showed that simultaneous inhibition of mTORC2 during everolimus treatment enhanced the antitumor effect of everolimus and prevented clear cell carcinoma cells of the ovary from acquiring resistance to RAD001 [25]. Furthermore, several reports have been published describing a feedback loop between the mTOR pathway and RAS/MAPK/ERK signaling, leading to activation of a different prosurvival signaling pathway upon mTOR inhibition. This mechanism of action suggests that treatment with everolimus as a single molecular target agent may not be sufficient, emphasizing the interest of studies with everolimus combined with other targeted agents [6, 14, 26].

In conclusion, given the high rate of clinical benefit and the relatively low toxicity profile found in this MTC subgroup analysis, we believe that further investigation in larger cohorts of MTC patients is now warranted, using everolimus either as a single agent or in sequential or combination therapy.

## Supplementary Material

Results of somatic mutation hotspot analysis. One MEN2A patient carried a germline *RET* c.1858T>C, p.Cys620Arg mutation. Four tumors showed a somatic *RET* c.2753T>C, p.Met918Thr mutation, including one that also carried an *EGFR* c.2543C>T, p.Pro848Leu mutation.

## Figures and Tables

**Table 1:** Baseline characteristics.

	All patients (*n* = 7)
Gender (*n*, %)	
Female	2 (29)
Male	5 (71)
Age (year; median, range)	53 (44–74)
Time from diagnosis (year; median, range)	4.3 (1.6–25.6)
Initial TNM stage (*n*, %)	
IB (T2 N0 M0)	0
IIB (T2-3 N0-1 M0)	2 (29)
IIIA (T1-3 N1-2 M0)	3 (43)
IV (any T any N M1)	2 (29)
Unknown	0
Tumor extent at study entry (*n*, %)	
Locally advanced	1 (14)
Metastatic	6 (86)
Number of disease sites (*n*, %)	
1	1 (14)
2	2 (29)
≥3	4 (57)
Mutational status (*n*, %)	
RET M918T	4 (57)
MEN-IIA	1 (14)
EGFR P848L	1 (14)
Unknown	1 (14)
Prior treatment (*n*, %)	
Surgery	6 (86)
Radiation therapy	2 (29)
Tyrosine kinase inhibitor^x*˚*^	4 (57)

^x^3 patients received XL184; 1 patient showed an ongoing PR for 14 months before he became progressive; 2 had PD as best result after 12 and 24 weeks. °1 patient had vandetanib for 8 months, followed by 3 months of sunitinib, which was stopped due to side effects.

**Table 2:** Efficacy analysis.

Parameter	
Median duration of treatment (weeks; range)	17 (6–116)
Cumulative dose of everolimus (mg; median, range)	1200 (440–8012)
Median duration of follow-up (weeks; range)	28 (17–147)
Best response by RECIST 1.0 (*n*, %)	
Complete response	0 (0)
Partial response	0 (0)
Stable disease	5 (71)^†^
Progressive disease	2 (29)
Overall disease control	5 (71)
Median duration of SD (weeks; range)	24 (17–116)^‡^
Median PFS (weeks; 95% CI)	33 (8–56)
Median OS (weeks; 95% CI)	30 (15–45)

RECIST: Response Evaluation Criteria in Solid Tumors, SD: stable disease, PFS: progression-free survival, and OS: overall survival.

^†^4 of 5 patients showed SD >24 weeks; ^‡^at time of data analysis, 1 patient still had ongoing SD.

**Table 3:** Changes in calcitonin and CEA during treatment.

Patient		Level at baseline	Maximum reduction (%)	Level at study exit	Increase from baseline till study exit (%)	Best response
1	CT (pmol/L)	73900	—	77700	105,1	SD
	CEA (*µ*g/L)	246,5	—	353,3	143,3	

2	CT (pmol/L)	245	—	280	114,3	SD
	CEA (*µ*g/L)	354,3	—	2208,0	623,2	

3	CT (pmol/L)	6750	—	29800	441,4	PD
	CEA (*µ*g/L)	1477,0	—	2081,0	140,9	

4	CT (pmol/L)	12716	30,3	8869	‡	PD
	CEA (*µ*g/L)	216,9	4,9	247,9	114,3	

5	CT (pmol/L)	680	37,3	1148	168,8	SD
	CEA (*µ*g/L)	117,1	—	164,4	140,4	

6	CT (pmol/L)	58	56,9	89	153,4	SD
	CEA (*µ*g/L)	70,7	35,5	86,7	122,6	

7	CT (pmol/L)	2156	—	5239^+^	243^+^	SD
	CEA (*µ*g/L)	91,1	10,1	166,7^+^	183^+^	

CT: calcitonin and CEA: carcinoembryonic antigen level.

—: there was no reduction in CT or CEA compared to baseline and ‡: there was no increase in CT compared to baseline; ^+^patient is still on everolimus treatment.

**Table 4:** Adverse events.

Event	All	Grades number of patients (% of category)			
	Number of patients (% of total (*n* = 7))	1	2	3	4
Mucositis	3 (43)	2 (67)	1 (33)		
Fatigue	3 (43)	1 (33)		2 (67)	
Hypertriglyceridemia	3 (43)	1 (33)	2 (67)		
Peripheral edema	2 (29)		1 (50)	1 (50)	
Anorexia	2 (29)	2 (100)			
Diarrhea	2 (29)	1 (50)	1 (50)		
Rash	2 (29)	1 (50)	1 (50)		
Pneumonia	2 (29)	1 (50)		1 (50)	
Liver function disorders	2 (29)	1 (50)	1 (50)		
Hypercholesterolemia	2 (29)	1 (50)		1 (50)	
Hypophosphatemia	2 (29)	2 (100)			
Hypoparathyroidism	2 (29)	2 (100)			
Weight loss	1 (14)	1 (100)			
Nausea	1 (14)	1 (100)			
Vomiting	1 (14)	1 (100)			
Constipation	1 (14)	1 (100)			
Allergic reaction	1 (14)		1 (100)		
Dry skin	1 (14)	1 (100)			
Itch	1 (14)		1 (100)		
Asthenia	1 (14)	1 (100)			
Anemia	1 (14)		1 (100)		
Hyperglycemia	1 (14)			1 (100)	
Cough	1 (14)		1 (100)		
Dyspnea	1 (14)	1 (100)			
Pneumonitis	1 (14)			1 (100)	

All AEs graded according to Common Terminology Criteria for Adverse Events version 4.0.

**Table 5:** Summary of everolimus pharmacokinetic parameters.

	Median (range)	Mean	SD	CV%
AUC_0–24 hr_ (*µ*g*∗*hr/L)	421 (257–959)	442	246	55.7%
*C*_trough_ (*µ*g/L)	7.4 (4–13.8)	9.0	5.2	57.8%
*T*_max⁡_ (hr)	1 (0.33–3.08)	1.2	0.9	75.0%
*C*_max⁡_ (*µ*g/L)	48.2 (37.8–102.3)	59.4	23.8	40.1%
*T*_1/2_ (hours)	13.8 (10.9–32.4)	16.5	7.5	45.5%

AUC is area under the concentration time curve, *C*_max⁡_ is peak plasma concentration, CV% is coefficient of variation, SD is standard deviation, and *T*_max⁡_ is time to reach peak plasma concentration.
