# Supplementary material for: Beneficial Effects of the mTOR Inhibitor Everolimus in Patients with Advanced Medullary Thyroid Carcinoma: Subgroup Results of a Phase II Trial
Source: Int J Endocrinol. 2015 Jul 29;2015:348124. doi: 10.1155/2015/348124 (PMC4532868; doi:10.1155/2015/348124)
Supplement: Supplementary file 1 — Results of somatic mutation hotspot analysis. One MEN2A patient carried a germline RET c.1858T>C, p.Cys620Arg mutation. Four tumors showed a somatic RET c.2753T>C, p.Met918Thr mutation, including one that also carried an EGFR c.2543C>T, p.Pro848Leu mutation. [file 348124.f1.pdf]

Supplementary Table 1 Somatic mutations

Sample ID	Gene	Chromosome	Chromosome position	Exon	Coverage	Mutation Frequency (%)		AA change	Co
**MTC29**	RET	10	43617416	16	85	21,18	c.2753T>C	p.Met918Thr	CO
**MTC31**	RET	10	43617416	16	1751	64,13	c.2753T>C	p.Met918Thr	CO
**MTC33**	RET	10	43617416	16	606	64,13	c.2753T>C	p.Met918Thr	CO
**MTC34**	EGFR	7		21	2093	51,84	c.2543C>T	p.Pro848Leu	CO
**MTC34**	RET	10	43617416	16	647	50,85	c.2753T>C	p.Met918Thr	CO
**MTC34**	RET	10	43609101	10			c.1858T>C	p.Cys620Arg	CO

The reference sequences used were NM_020975.4 for RET and NM_005228 for EGFR
